# Influence of Ag@SiO_2_ with Different Shell Thickness on Photoelectric Properties of Hole-Conductor-Free Perovskite Solar Cells

**DOI:** 10.3390/nano10122364

**Published:** 2020-11-27

**Authors:** Zhiyuan He, Chi Zhang, Rangwei Meng, Xuanhui Luo, Mengwei Chen, Haifei Lu, Yingping Yang

**Affiliations:** School of Science, Wuhan University of Technology, Wuhan 430070, China; hezhiyuan960705@whut.edu.cn (Z.H.); zhangchi13986286672@whut.edu.cn (C.Z.); mengrangwei@whut.edu.cn (R.M.); luoxuanhui@whut.edu.cn (X.L.); mengwei.chen@whut.edu.cn (M.C.); haifeilv@whut.edu.cn (H.L.)

**Keywords:** Ag@SiO_2_ core-shell nanoparticles, perovskite solar cells, localized surface plasmon resonance effect, scattering effect, perovskite solar cells

## Abstract

In this paper, Ag@SiO_2_ core-shell nanoparticles (NPs) with different shell thicknesses were prepared experimentally and introduced into the photosensitive layer of mesoscopic hole-conductor-free perovskite solar cells (PSCs) based on carbon counter electrodes. By combining simulation and experiments, the influences of different shell thickness Ag@SiO_2_ core-shell nanoparticles on the photoelectric properties of the PSCs were studied. The results show that, when the shell thickness of 0.1 wt% Ag@SiO_2_ core-shell nanoparticles is 5 nm, power conversion efficiency is improved from 13.13% to 15.25%, achieving a 16% enhancement. Through the measurement of the relevant parameters of the obtained perovskite film, we found that this gain not only comes from the increase in current density that scholars generally think, but also comes from the improvement of the film quality. Like current gain, this gain is related to the different shell thickness of Ag@SiO_2_ core-shell nanoparticles. Our research provides a new direction for studying the influence mechanism of Ag@SiO_2_ core-shell nanoparticles in perovskite solar cells.

## 1. Introduction

Perovskite solar cells (PSCs) have low temperature solution processability, high defect tolerance, large light-harvesting coefficient, low composite material loss, long carrier diffusion distance, adjustable band gap, and other excellent properties [1,2,3,4,5,6] and have received widespread attention. Up to now, the power conversion efficiency (PCE) of PSCs increased from 3.8% to 25.5% [7,8].

Despite great progress in the photovoltaic conversion efficiency of inorganic–organic hybrid (PSCs) having been achieved, the large-scale application of PSCs still faces serious challenges due to the poor-stability and high-cost of the spiro-OMeTAD hole transport layer [9]. It was found that hole-conductor-free PSCs can also operate efficiently due to the unique ambipolar property of the perovskite, which allows them to serve not only as a light harvester but also as a hole conductor [4]. However, the photovoltaic conversion efficiency of hole-conductor-free PSCs is still lower [9]. One effective approach to improve photovoltaic conversion efficiency of PSCs is to introduce noble metallic nanoparticles into their structure, such as a hole transport layer, an electron transport layer, and a perovskite layer. Noble metallic nanoparticles could limit resonant photons and form intense near-field electromagnetic fields, thereby greatly enhancing their light absorption and scattering properties [10,11,12,13]. By adjusting the shape, particle size, concentration, and other conditions of precious metal nanomaterials, the peak position and strength of their localized surface plasmon resonance (LSPR) can be changed to meet the requirements of PSCs in different environments [14]. Introducing noble metallic nanomaterials can not only improve the optical absorption of PSCs, but also improve the electrical property of the PSCs [15]. At the same time, noble metallic nanomaterials can reduce the thickness of the photosensitive layer by increasing the light absorption of the PSCs, thereby reducing the toxicity of PSCs [16].

According to the results of theoretical simulation, the closer the distance between the noble metallic nanomaterials and the photosensitive layer, the stronger localized surface plasmon enhancement can be obtained by the photosensitive layer [17]. However, due to the strong corrosiveness of CH_3_NH_3_PbI_3_, the noble metallic nanomaterials will be corroded if the noble metallic nanomaterials are in direct contact with CH_3_NH_3_PbI_3_. In addition, their direct contact will create charge recombination sites resulting in the reduction of PSCs performance. Therefore, in previous research, the applications of metal nanoparticles in PSC were majorly embedded in three layers: the mesoporous layer [18,19,20,21,22,23], the hole transport layer [24,25,26,27,28,29], and the electron transport layer [30,31,32,33,34,35]. For example, in 2016, Nourolahi et al. introduced Ag nanoparticles with different concentrations into the mesoporous TiO_2_ layer of PSCs without the hole transport layer, which can increase the power conversion efficiency by more than 30%. The test found that the improved current is not only caused by LSPR and scattering effects, but also caused by the reduction of carrier recombination. At the same time, the author calculated Deff (electron diffusion coefficients), electron transit time, and other data that showed that the improvement of its electrical property was mainly achieved by reducing the electron transit time and reducing carrier recombination [30].

To solve the corrosion of noble metallic nanomaterials by CH_3_NH_3_PbI_3_, some scholars chose to wrap noble metal nanoparticles with a protective layer and then introduced them into the photosensitive layer to enhance property of PSCs and reduced their influence on stability [35,36,37]. In 2019, Deng et al. successfully embedded Au@Ag@SiO_2_ core-shell nanocuboids in the photosensitive layer of planar heterojunction PSCs, increasing the PCE from 15.41% to 17.38% [35]. At the same time, they found that introducing Au@Ag@SiO_2_ core-shell nanocuboids can also improve the film formation quality of the photosensitive layer, but they did not find the source of this gain.

Up to now, few scholars have studied the influence of the thickness of the protective layer of core-shell materials on PSCs’ photoelectric properties. In this work, Ag@SiO_2_ core-shell nanoparticles with a different shell thickness were introduced into the active layer of the PSCs, and the influences of Ag@SiO_2_ core-shell nanoparticles with different shell thickness on the photoelectric properties of the PSCs were discussed through experiments and simulations. An increment in power conversion efficiency (PCE) of 16%, from 13.13% to 15.25%, could be obtained after the addition of Ag@SiO_2_ core-shell nanoparticles.

## 2. Materials and Methods

### 2.1. Ag NPs Preparation

In this paper, the silver ions in silver nitrate (AgNO_3_) were changed to Ag nanoparticles by using ethylene glycol. In the reaction process, polyvinylpyrrolidone (PVP) was used as a protective agent, and then an insulating SiO_2_ shell layer was wrapped on the surface of Ag nanoparticles by the improved Stober method to obtain Ag@SiO_2_ core-shell nanoparticles (NPs).

The specific experimental process is as follows. First, 3 g of PVP powder were taken and dissolved in 40 mL of ethylene glycol and 0.5 g of AgNO_3_ crystals were taken and dissolved in 20 mL of ethylene glycol. After dissolution, the ethylene glycol solution of PVP was put into a thermostatic magnetic stirrer and heated while stirring. When the temperature rose to 120 °C, the ethylene glycol solution of AgNO_3_ was added to it dropwise and the resulting solution was kept at 120 °C for 1 h under stirring. After the solution was taken out and cooled to room temperature, moderate deionized water was added to the solution, which was followed by centrifugation. Finally, the precipitate was washed with deionized water and acetone several times, and then dried at 40 °C to obtain the Ag NPs.

### 2.2. Ag@SiO_2_ NPs Preparation

First, 120 mg of Ag nanoparticles were dispersed in 120 mL of ethanol by ultrasound for 30 min. After ultrasonic dissolution, the solution was placed on a magnetic stirrer and kept under stirring. Second, 2.5 mL ammonia, 2.5 mL deionized water, and 0.1 mL (0.15 mL, 0.2 mL, 0.25 mL, and 0.3 mL) tetraethyl orthosilicate (control the thickness of the SiO_2_ shell of the Ag@SiO_2_ core-shell nanoparticles generated by adjusting the amount of tetraethyl orthosilicate) were added to it. Finally, the solution was stirred for 12 h at room temperature and Ag@SiO_2_ NPs were obtained by centrifugation, washing (with ethyl alcohol several times), and desiccation.

### 2.3. Preparation of the Device

First, the fluorine-doped tin oxide (FTO) conductive glasses were cleared by a soft brush, and then was ultrasonically cleaned with detergent, acetone, and isopropanol for 30 min. The cleaned FTO conductive glasses were placed in a beaker filled with clean ethanol, sealed, and placed in a dark place to reserve.

Second, the precursor solution of the dense layer of TiO_2_ was prepared by adding 0.1 mL of titanium diisopropoxide to 1.9 mL of ethanol and stirred for one hour. The precursor solution of the mesoporous layer was obtained by adding colloidal solutions of ZrO_2_ and TiO_2_ to ethanol at a mass ratio of 1:5 and stirred for 12 h.

Third, in the glove box, 462 mg of PbI_2_, 178 mg of CH_3_NH_3_I, 78 mg of dimethyl sulfoxide (DMSO), and 600 mg of dimethylformamide (DMF) (mixed Ag@SiO_2_ core-shell nanoparticles (0.1 wt%)) were added to the brown vial. The brown vial was placed on a magnetic stirrer and continuously stirred for about 4 h.

Fourth, the cleaned FTO glass was blown dry, and then placed in a UV ozone cleaner for 30 min of light cleaning. The dense layer precursor solution was spin-coated on the FTO at a speed of 4000 rpm for 20 s and annealed at a temperature of 150 °C for 10 min. After cooling, the above steps were repeated, and then annealed at 500 °C for 30 min to form a dense film. The TiO_2_ mesoporous layer was coated on the top of the dense layer by spin-casting at 3500 rpm for 20 s, and then annealed at 150 °C for 10 min, which was followed by annealing at 500 °C for 30 min. After cooling to room temperature, the ZrO_2_ mesoporous layer was applied in the same way (change the speed to 5000 rpm). The perovskite layer was formed by spin-coating the precursor solution (35 µL) at 1000 rpm for 10 s and 4000 rpm for 30 s, which was followed by annealing at 100 °C for 10 min. During 5 s at 4000 r/min, about 180 µL of methylbenzene were dropped on the spinning substrates to ensure the fast crystallization of perovskite by extracting the solvent of the perovskite precursor solution.

Finally, the FTO conductive glasses were placed under the screen-printing plate and scraped carbon onto the FTO, which was followed by annealing at 100 °C for 10 min. So far, the entire PSC was completed.

### 2.4. Instrumentation and Characterization

The TEM images of Ag@SiO_2_ core-shell nanoparticles were measured by a JEM-2010 FEF transmission electron microscope at an acceleration voltage of 200 KV. X-Ray diffractometer (XRD) images were acquired from an X-ray diffractometer (Advance D8, AXS, Rigaku Corporation, Tokyo, Japan). The SEM images of the surface and cross section of the prepared sample were scanned by a field emission scanning electron microscope (JSM-IT300, JEOL Ldt, Tokyo, Japan). The light-absorption spectra were obtained via UV-vis spectrophotometry (UV3600, Shimadzu Corporation, Tokyo, Japan). The photocurrent-voltage (J-V) characteristics were tested by solar simulator (Oriel Sol3A, Newport Corporation, Irvine, CA, USA) under AM 1.5G illumination at 100 mW/cm^2^ intensity. Incident photon-to-electron conversion efficiency (IPCE) (Newport Corporation, Irvine, California, USA) was used to investigate the quantum efficiency of the PSC devices. The photoluminescence (PL) spectroscopy of PSCs was tested by fluorescence spectrometer (RF-6000, Shimadzu Corporation, Tokyo, Japan). The electrochemical impedance spectroscopy (EIS) of PSCs was obtained by an electrochemical workstation (Zahner Company, Kronach, Germany) for frequencies of 10 mHz to 10 MHz at a bias of 0.8 V under simulated AM 1.5 G radiation (irradiance of 100 mW/cm^2^) with an alternating current (AC) signal amplitude of 10 mV at room temperature.

### 2.5. Simulation

In this paper, the simulation was mainly based on the finite difference time domain (FDTD) method proposed by Kane Yee in 1966 [38]. The core idea was to use the central difference to express the Maxwell curl equation with time variables in the difference form, and to convert the differential with continuous variables. The equation was converted into a different equation with a finite number of unknowns. By replacing the continuous electromagnetic field space with the FDTD basic unit, the problem in the continuous electromagnetic field space was transformed into a numerical solution problem at each discrete point.

**Figure 1:**
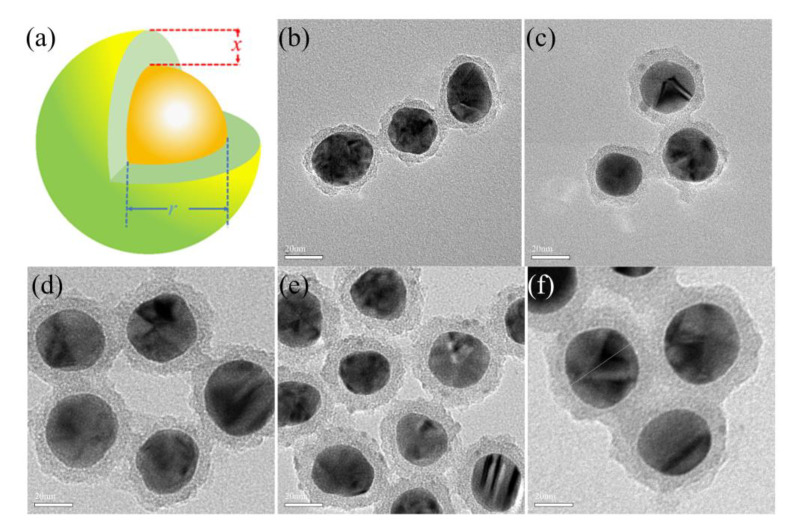
(**a**) Schematic diagram of Ag@SiO_2_ core-shell nanoparticle structure. TEM images of Ag@SiO_2_ core-shell nanoparticles with different shell thickness: (**b**) 3 nm, (**c**) 5 nm, (**d**) 7 nm, (**e**) 9 nm, and (**f**) 11 nm.

The schematic diagram of the Ag@SiO_2_ core-shell nanoparticles is shown in Figure 1a. The simulation results of the PSCs introduced with Ag@SiO_2_ core-shell nanoparticles with different shell thickness were obtained by keeping the radium (18 nm) of the internal silver core Ag unchanged and adjusting the thickness x of the shell layer SiO_2_. The simulation wavelength was set to 300–800 nm, the core Ag radius of Ag@SiO_2_ core-shell nanoparticles was 18 nm, and the shell thickness was 3 nm, 5 nm, 7 nm, 9 nm, and 11 nm. The refractive index of each material comes from the Refractive index database.

## 3. Results and Discussion

In this paper, the preparation method, the selection of the diameter, and concentration of the silver core is all from the literature that have been reported [39]. Through the transmission electron microscopy (TEM), the shell thickness of Ag@SiO_2_ core-shell nanoparticles under different ratios has clear changes (Figure 1). The diameter of the silver core is mainly distributed in the range of 20–40 nm. The thickness of the shell changes with the amount of tetraethyl orthosilicate (0.1 mL, 0.15 mL, 0.2 mL, 0.25 mL, and 0.3 mL), which are about 3 nm (Figure 1b), about 5 nm (Figure 1c), 7 nm (Figure 1d), 9 nm (Figure 1e), and 11 nm (Figure 1f), respectively.

**Figure 2:**
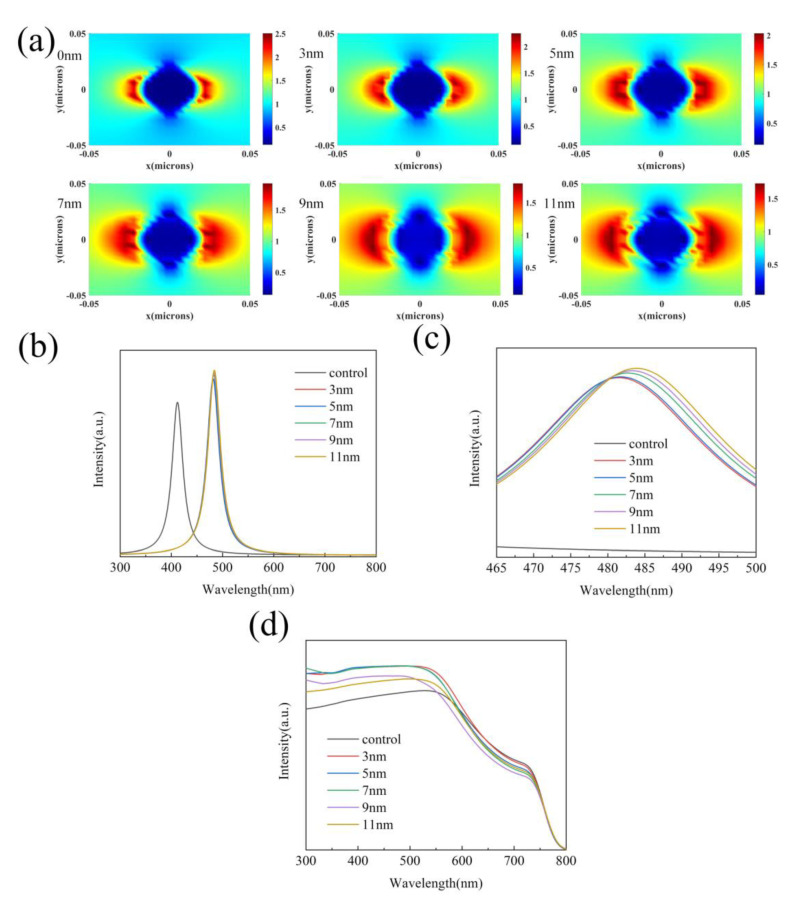
(**a**) Local field simulation of Ag@SiO_2_ nanoparticles with a different shell thickness. (**b**) Scattering spectra of Ag@SiO_2_ nanoparticles with a different shell thickness. (**c**) Local amplified scattering spectra of Ag@SiO_2_ nanoparticles with a different shell thickness. (**d**) Light absorption spectrum of CH_3_NH_3_PbI_3_ films with Ag@SiO_2_ core-shell nanoparticles with a different shell thickness.

In 1957, American scientists first proposed surface plasmons [40], and it was confirmed two years later [41]. As stated in the theory, when Ag@SiO_2_ core-shell nanoparticles are added to PSCs, the frequency of the external electric field matches the frequency of the collective oscillation of free electrons. Surface plasmon will be generated on the silver nanoparticle surface, which improves the capture of photons, which improves the PSC’s absorption of light. At the same time, it will significantly enhance the electromagnetic field on the surface of Ag@SiO_2_ core-shell nanoparticles. Therefore, we can use the intensity of the local field on the surface of the nanoparticles to characterize the intensity of the LSPR effect of the nanoparticles. Ag@SiO_2_ core-shell nanoparticles also have a scattering effect. When light is irradiated into the PSCs, it can extend the light path to improve the capture of photons of PSCs [42]. To evaluate the changes of the two effects when the shell thickness changes, we obtained the local field (Figure 2a) and scattering spectra (Figure 2b) of Ag@SiO_2_ nanoparticles with different shell thicknesses through simulations. As shown in Figure 2a, when the thickness of the shell layer increases, the electric field intensity on the surface of the nanoparticle shows a downward trend, which indicates that the raise of the shell layer thickness will reduce the intensity of the LSPR effect. Figure 2b,c show that, as the thickness of the shell layer increases, the scattering peak of the nanoparticle has a red shift and the intensity improves. The former is because SiO_2_ has a larger refractive index, which causes the peak position to shift, while the latter is because the increased thickness increases the scattering cross section of the nanoparticles to improve the scattering effect. From the above discussion, it can be concluded that the change of the shell thickness has the opposite effect on the LSPR effect and the scattering effect. To further study the combined influence of the two effects, the absorption spectrums of the perovskite films of Ag@SiO_2_ nanoparticles with different shell thicknesses were measured in Figure 2d. It can be seen that the raise in the thickness of the shell layer will reduce the light absorption of the film, which shows that SiO_2_ that is too thick is not conducive to the light absorption of the active layer.

**Figure 3:**
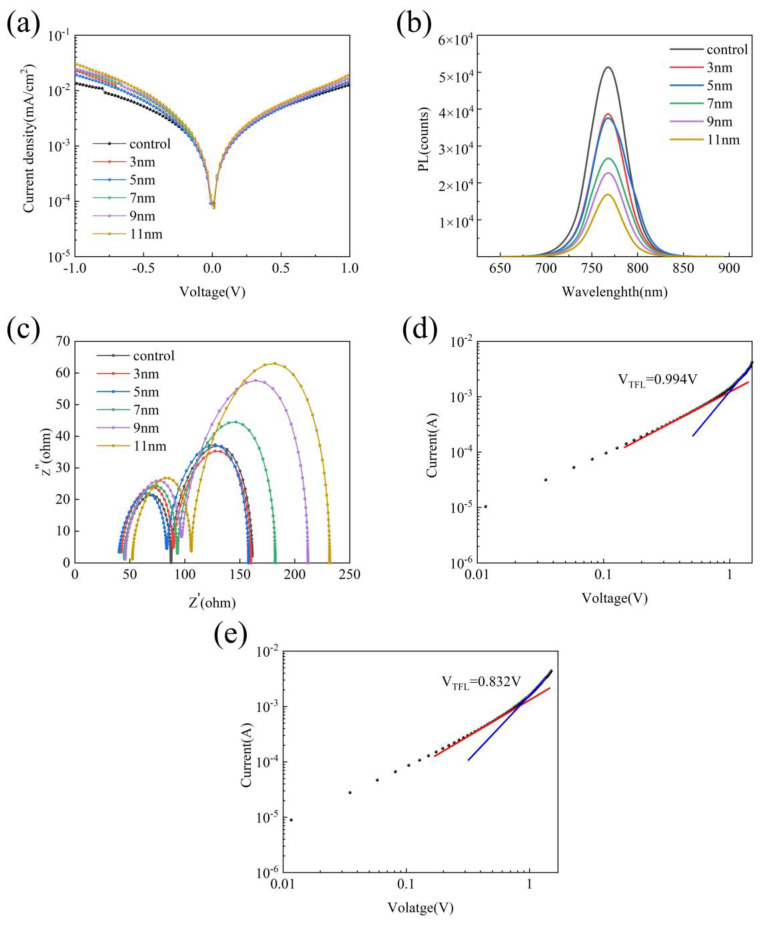
(**a**) Dark current of the FTO/perovskite/carbon structure PSCs with Ag@SiO_2_ core-shell nanoparticles with a different shell thickness. (**b**) PL spectra and (**c**) EIS spectra of PSCs with Ag@SiO_2_ core-shell nanoparticles with different shell thicknesses. (**d**) Current-voltage of FTO/perovskite/carbon structure pristine PSCs. (**e**) Current-voltage of FTO/perovskite/carbon structure PSCs with Ag@SiO_2_ core-shell nanoparticles with a 5-nm shell thickness.

It has been reported that adding noble metallic nanomaterials to PSCs can not only increase the light absorption of the PSCs, but also can reduce the recombination of carriers to enhance the mobility of carriers [16]. In 2015, Saliba et al. proposed a theory whereby the radiative dipoles of the excitons (electron–hole pairs shortly proceeding recombination) within the perovskite film interacting with the dipoles of the metal nanoparticles. This interaction results in enhanced (faster) radiative decay but also partially guides the reemitted light into the plane of the film. The first mechanism increases the external photoluminescence, but the second mechanism reduces the external photoluminescence by increasing the reabsorption of emitted light within the perovskite film [22]. From this theory, when the thickness of the shell increases, the internal Ag core is far away from radiative dipoles of the excitons in the perovskite film, reducing the radiative decay. Meanwhile, the increased shell thickness can reabsorb more light collected back into the PSCs. The carrier recombination rate will reduce under the combined action of these two mechanisms. To confirm this inference, the dark current spectra of PSCs with Ag@SiO_2_ core-shell nanoparticles with different shell thickness were tested under dark conditions (Figure 3a). It can be found that, as the thickness of the shell increases, the PSCs’ dark current shows a downward trend. It indicates that, when the thickness of the shell increases, carrier recombination will decrease. To further prove this conclusion, the steady-state PL spectroscopies of the perovskite layers added with Ag@SiO_2_ core-shell nanoparticles with different shell thickness were measured by the fluorescence spectrometer (Figure 3b). As shown in Figure 3b, as the thickness of the shell of Ag@SiO_2_ core-shell nanoparticles raises, the intensity of the PL peak shows a downward trend. It indicates that the recombination of carrier falls with the increase of the shell thickness of Ag@SiO_2_ core-shell nanoparticles. In addition, the EIS analyses were performed for frequencies of 10 mHz to 10 MHz at a bias of 0.8 V under simulated AM 1.5G radiation (irradiance of 100 mW/cm^2^) to further understand charge transport and charge recombination in the devices (Figure 3c). As shown in Figure 3c, as the thickness of the shell layer increases, the recombination resistance (R_rec_) increases while the series resistance (R_s_) and the transmission resistance (R_tr_) first increase and then decrease. The higher R_rec_ indicates that the recombination of excitons in the PSCs decreases as the thickness of the shell increases, which is also consistent with the PL spectra. Based on the above analysis, it can be concluded that Ag@SiO_2_ core-shell nanoparticles are added into the perovskite layer of a PSCs. The suitable Ag@SiO_2_ nanoparticles can decrease carrier recombination, and the gain will increase as the thickness of the shell grows.

Furthermore, we prepared the FTO/perovskite/carbon structure of PSCs with an Ag@SiO_2_ core-shell nanoparticle with a shell thickness of 5 nm and pristine PSCs and measured their J-V curves (Figure 3d,e). The trap filling limit voltage (V_TFL_) can be obtained by Figure 3d,e and the trap density of the two PSCs can be calculated by Equation (1).

(1) $$N_{trap}=2\varepsilon_{0}\varepsilon_{r}V_{TFL}/qd^{2}$$

where N_trap_ is the trap density, ε_0_ is the vacuum dielectric constant, ε_r_ is the relative dielectric constant of CH_3_NH_3_PbI_3_, V_TFL_ is the trap filling limit voltage, q is the charge of the elementary charge, and d is the thickness of the perovskite film [43].

It can be calculated that the trap density of the perovskite solar cell (PSC) with Ag@SiO_2_ core-shell nanoparticles with 5 nm thickness of the shell is reduced from 1.97 × 10^18^ cm^−3^ to 1.65 × 10^18^ cm^−3^. It shows that the Ag@SiO_2_ core-shell nanoparticles can improve the quality of perovskite film through passivating defects.

**Figure 4:**
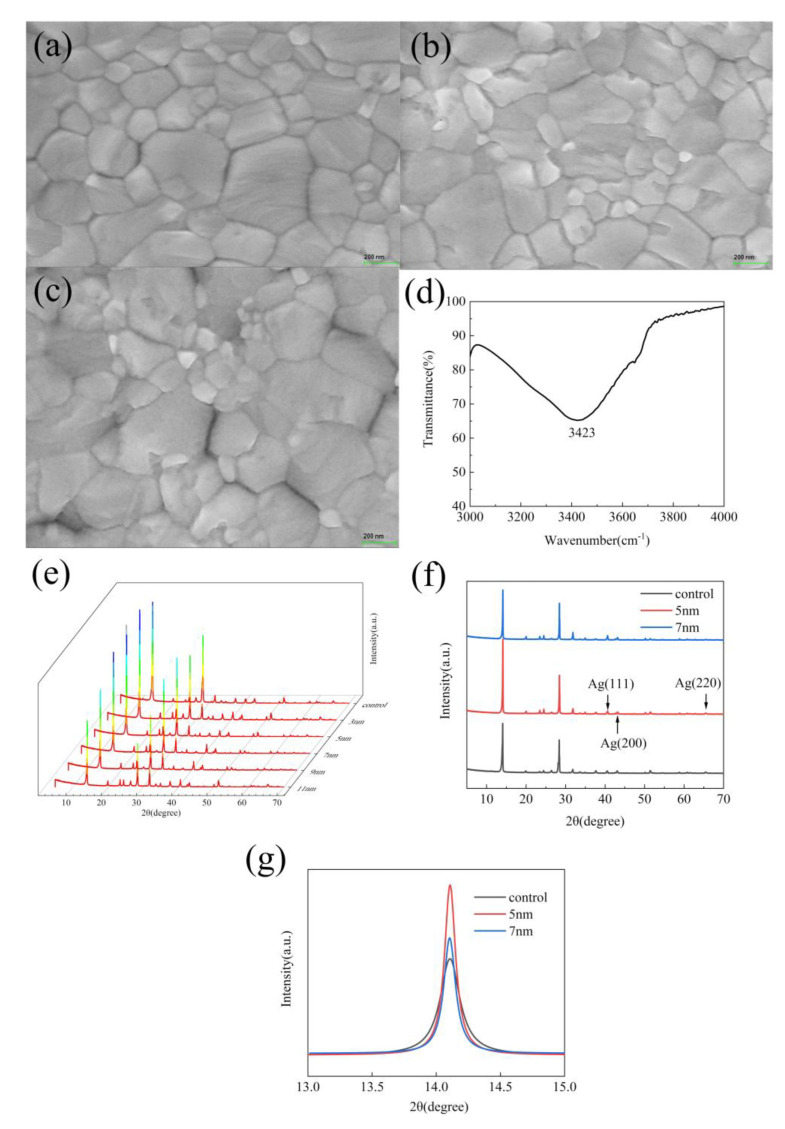
SEM images of (**a**) pristine CH_3_NH_3_PbI_3_ film. (**b**) CH_3_NH_3_PbI_3_ films with a 5-nm shell thickness of Ag@SiO_2_ core-shell nanoparticles. (**c**) CH_3_NH_3_PbI_3_ films with an 11-nm shell thickness of Ag@SiO_2_ core-shell nanoparticles. (**d**) Fourier Transform Infrared Spectrometer of nanoparticles. (**e**) XRD patterns of the pristine CH_3_NH_3_PbI_3_ film and CH_3_NH_3_PbI_3_ films with 3 nm, 5 nm, 7 nm, 9 nm, and 11 nm shell thickness of Ag@SiO_2_ core-shell nanoparticles. (**f**) XRD patterns of the pristine CH_3_NH_3_PbI_3_ film, CH_3_NH_3_PbI_3_ films with a 5-nm shell thickness Ag@SiO_2_ core-shell nanoparticles, and CH_3_NH_3_PbI_3_ films with a 7-nm shell thickness of Ag@SiO_2_ core-shell nanoparticles. (**g**) XRD patterns of detailed view of the (110) diffraction peak ranging from 13° to 14°.

To explore the effect of the thickness of the shell on the quality of the film, SEM images and XRD images were tested. Figure 4a shows the SEM image of the pristine CH_3_NH_3_PbI_3_ film, where big gaps between the perovskite grains can be observed. After adding 5 nm and 11 nm shell thickness of Ag@SiO_2_ core-shell nanoparticles into the *N,N*-dimethylformamide (DMF) precursor solution, the different CH_3_NH_3_PbI_3_ film (Figure 4b,c) can be gained. As shown in Figure 4b, there are more compact crystal grains and smaller gaps than those of the pristine CH_3_NH_3_PbI_3_ film. When the shell thickness of Ag@SiO_2_ core-shell nanoparticles was further increased to 11 nm (Figure 4c), the grain size of CH_3_NH_3_PbI_3_ is sharply decreased. The crystallization of CH_3_NH_3_PbI_3_ is clearly damaged. This indicates that adding suitable Ag@SiO_2_ core-shell nanoparticles can improve the CH_3_NH_3_PbI_3_ film quality of the PSCs. This improvement can be attributed to the Ag@SiO_2_ core-shell nanoparticles whose outer shell SiO_2_ is produced by the hydrolysis of tetraethyl orthosilicate. Therefore, their surfaces have many hydroxyl groups. As shown in Figure 4d, the broad absorption around 3300 cm^−1^ was assigned to the Si–OH residue, formed on hydrolysis of alkoxy groups of tetraethyl orthosilicate [44]. When the number of hydroxyl groups is small, these hydroxyl groups can bond with the uncoordinated Pb^2+^ in CH_3_NH_3_PbI_3_, so that strong and stable interactions between the perovskite and Ag@SiO_2_ core-shell nanoparticles can be generated, which can improve the crystallinity of CH_3_NH_3_PbI_3_. However, the oversize Ag@SiO_2_ core-shell nanoparticles in the PbI_2_ film would lead to greater and faster nucleation of PbI_2_ and inhibition of PbI_2_ crystal growth [45]. It has been reported that PbI_2_ acts as a framework and provides “nucleation” centers for the subsequent crystallization of the perovskite film [46,47]. Thus, when the number of hydroxyl groups is large, the nucleation of the perovskite accelerates and hinder the growth of the perovskite grain, resulting in the bad morphology of the perovskite film [45]. To further illustrate the influence of the incorporation of Ag@SiO_2_ core-shell nanoparticles with a different shell thickness on the quality of CH_3_NH_3_PbI_3_ film, we conducted XRD tests. As shown in Figure 4e, when the shell thickness of Ag@SiO_2_ core-shell increases, the XRD peak corresponding to (110) plane becomes first sharp and then broad. It indicates that, when the thickness of the shell layer is low, Ag@SiO_2_ core-shell nanoparticles can improve the formation quality of the CH_3_NH_3_PbI_3_ film. When the thickness of the shell layer is too high, it will reduce the formation quality of the CH_3_NH_3_PbI_3_ film. Figure 4f shows the XRD pattern of CH_3_NH_3_PbI_3_ without Ag@SiO_2_ core-shell nanoparticles and the XRD pattern with Ag@SiO_2_ core-shell nanoparticles. The strong diffraction peaks of the perovskite at 14.1°, 28.4°, 31.9°, and 40.7° can be observed in the XRD patterns of the CH_3_NH_3_PbI_3_ films without Ag@SiO_2_ core-shell nanoparticles. The same perovskite peaks can also be identified for CH_3_NH_3_PbI_3_ films with Ag@SiO_2_ core-shell nanoparticles. In addition, there are no extra peaks in the XRD curves of the CH_3_NH_3_PbI_3_ films with Ag@SiO_2_ core-shell nanoparticles, which means that the Ag@SiO_2_ core-shell nanoparticles do not affect the perovskite crystal structure and the Ag@SiO_2_ core-shell nanoparticles present in the CH_3_NH_3_PbI_3_ films are in the amorphous state. In the XRD pattern with Ag@SiO_2_ core-shell nanoparticles, several characteristic peaks of elemental Ag can be observed (38°, 44°, 65°). To further prove our conclusion, the detailed view and relevant information of the representative diffraction peak at the (110) facet are shown in Figure 4g and Table 1, respectively. Evidently, Ag@SiO_2_ core-shell nanoparticles were added into the perovskite structure, influencing the crystallinity and the size of perovskite grain, which coincides with the results of the SEM images above. Combining XRD patterns and space-charge-limited current (SCLC) tests, we can conclude that suitable Ag@SiO_2_ can improve the film quality by passivating defects.

**Figure 5:**
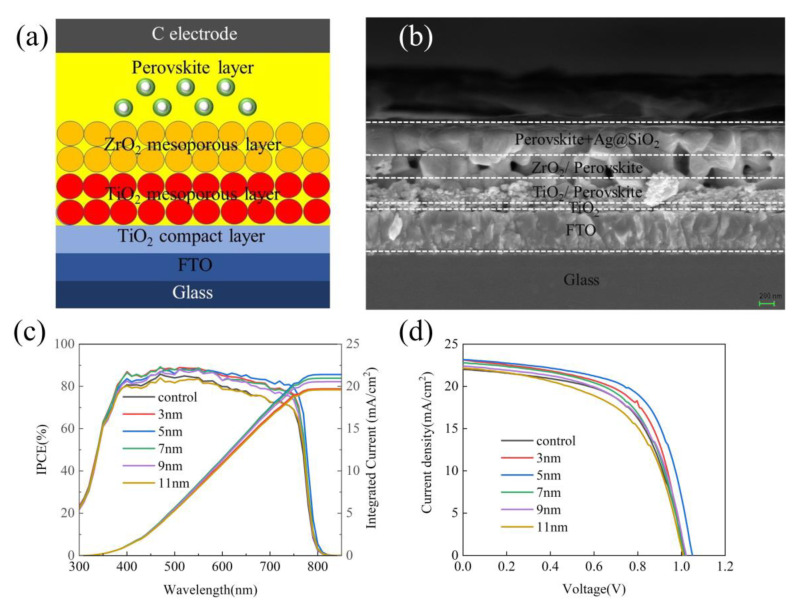
(**a**) Perovskite solar cells (PSC) schematic structure. (**b**) SEM image of a PSC cross section. (**c**) IPCE spectrum of PSCs with Ag@SiO_2_ core-shell nanoparticles with different shell thicknesses. (**d**) J-V curve of PSCs with Ag@SiO_2_ core-shell nanoparticles with different shell thicknesses.

Since Ag@SiO_2_ core-shell nanoparticles passivate defects, enhance photon absorption, and reduce carrier recombination and these effects vary with the thickness of the shell, it is important to find the best shell thickness to make the photoelectric properties of PSC reach the optimal value. As shown in Figure 5a, the schematic structure of PSC is the same as the SEM cross-sectional view of the PSC prepared (Figure 5b). An entire PSC with a TiO_2_ dense layer, a TiO_2_ mesoporous layer, a ZrO_2_ layer, and perovskite layer is fabricated onto a fluorine-doped tin oxide (FTO). The thickness of each layer from bottom to top was 500 nm, 40 nm, 150 nm, 150 nm, and 400 nm, and some perovskite material infiltrated the TiO_2_ mesoporous layer and the ZrO_2_ layer to form a composite layer. This is the same as that obtained in the previous literature [48]. Figure 5c shows that, as the shell thickness increases, the IPCE of the PSC first increases and then decreases. The initial enhancement is because, when the thickness of the shell is relatively thinner, the increase in the carrier mobility caused by the increase in the thickness of the shell is in an advantageous position in competition with the reduction in light absorption. When the shell thickness is relatively thicker, the promotion in carrier mobility caused by the increase in shell thickness is at a disadvantage in the competition with the reduction of light absorption. The results of the J-V curve (Figure 5d) also support this view.

**Figure 6:**
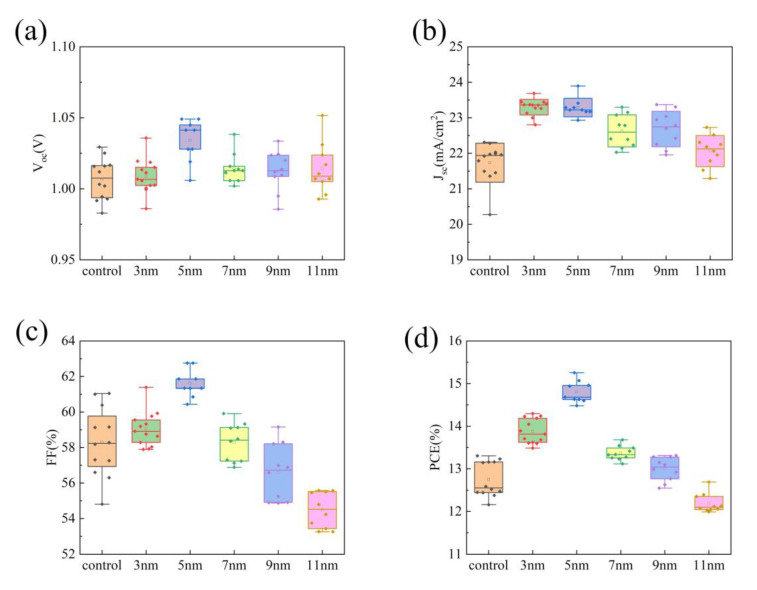
Box plots of PSCs’ photoelectric properties with Ag@SiO_2_ core-shell nanoparticles with a different shell thickness: (**a**) open circuit voltage, (**b**) short-circuit current value, (**c**) fill factor, and (**d**) photoelectric conversion efficiency.

The properties of PSCs with 0.1 wt% (from the reported literature) Ag@SiO_2_ core-shell nanoparticles with a different shell thickness were measured (Table 2) [39]. After comparing various properties of different PSCs, open circuit voltage (Figure 6a), short-circuit current density (Figure 6b), fill factor (Figure 6c), and photoelectric conversion efficiency (PCE) (Figure 6d). It can be found that, as the thickness of the shell layer increases, the open circuit voltage slightly improves. It is attributed that, when the thickness of the shell is thinner, the trap density of the PSCs is lower, which reduces the recombination of carriers at the perovskite/TiO_2_ interface. The short-circuit current density first increases and then decreases. The fill factor first increases and then decreases with the increase of the thickness of shell. This is because, when the thickness of the shell is relatively thinner, the trap density of the perovskite film is lower, and the recombination of carriers is less, thereby increasing the charge collection rate. When the thickness of the shell layer is relatively thicker, the trap density of the perovskite layer is too high, which affects the collection of charges. In general, adding 0.1 wt% Ag@SiO_2_ core-shell nanoparticles can effectively improve the photoelectric properties of PSCs, and when the thickness of the shell layer is about 5 nm, the gain reaches the highest. A 1.05 V of the open circuit voltage, a 23.17 mA/cm^2^ of the short-circuit current density, a 62.75% of a fill factor, and a 15.25% of the photoelectric conversion efficiency can be obtained.

## 4. Conclusions

In summary, adding Ag@SiO_2_ core-shell nanoparticles into the active layer of PSCs can increase the light absorption of the cell, reduce the recombination of carriers, and passivate surface defects, thereby improving the photoelectric properties of PSCs. When Ag@SiO_2_ nanoparticles with a mass ratio of 0.1 wt% and a shell thickness of 5 nm are added to the photosensitive layer of PSCs, the optimum PSCs can be obtained. The optimum PCE can improve from 13.14% to 15.25%. This means that adding Ag@SiO_2_ core-shell nanoparticles to the photosensitive layer of PSCs is an effective and efficient method to improve photoelectric properties of PSCs.

## Figures and Tables

**Table 1:** Relevant information of the representative diffraction peak at the (110) facet.

Sample	Control	3 nm	5 nm	7 nm	9 nm	11 nm
Intensity (a.u.)	15,774	18,035	24,963	16,828	11,820	9700
FWHM (°)	0.163	0.124	0.107	0.121	0.157	0.172

**Table 2:** Relevant properties parameters of the PSCs with Ag@SiO_2_ core-shell nanoparticles (NPs) with a different shell thickness.

Samples	V_oc_ (V)	J_sc_ (mA/cm^2^)	FF (%)	PCE (%)	R_s_ (Ω)	R_tr_ (Ω)	R_rec_ (Ω)	N_trap_ (cm^−3^)
Control	1.01	21.96	59.16	13.14	44.6	48.9	74.0	1.97 × 10^18^
3 nm	1.01	23.14	61.39	14.30	41.8	47.7	78.5	1.82 × 10^18^
5 nm	1.05	23.17	62.75	15.25	39.1	42.7	87.6	1.65 × 10^18^
7 nm	1.01	22.80	59.32	13.68	44.8	48.5	89	2.03 × 10^−6^
9 nm	1.02	22.26	58.30	13.28	45.7	51.3	115	2.16 × 10^18^
11 nm	1.03	21.96	53.26	12.06	52.3	53.5	126	2.31 × 10^18^
